# Supplementary material for: Machine Learning Approach to Select Small Compounds in Plasma as Predictors of Alzheimer’s Disease
Source: Int J Mol Sci. 2025 Jul 21;26(14):6991. doi: 10.3390/ijms26146991 (PMC12295269; doi:10.3390/ijms26146991)
Supplement: Supplementary file 1 [file ijms-26-06991-s001.zip › Supplementary Figures S1 and S2.pdf]

Supplementary Figures S1 and S2

Machine Learning Approach to Select Small Compounds in Plasma as Predictors of Alzheimer's Disease

Eleonora Stefanini [1], Alberto Iglesias [1], Joan Serrano-Marín [1], Juan Sánchez-Navés [2], Hanan A. Alkozi [3],
Mercè Pallàs [4,5,6], Christian Griñán-Ferré [4,5,6], David Bernal-Casas [7] and Rafael Franco [1,6,8,*]

Correspondence: rfranco123@gmail.com or rfranco@ub.edu

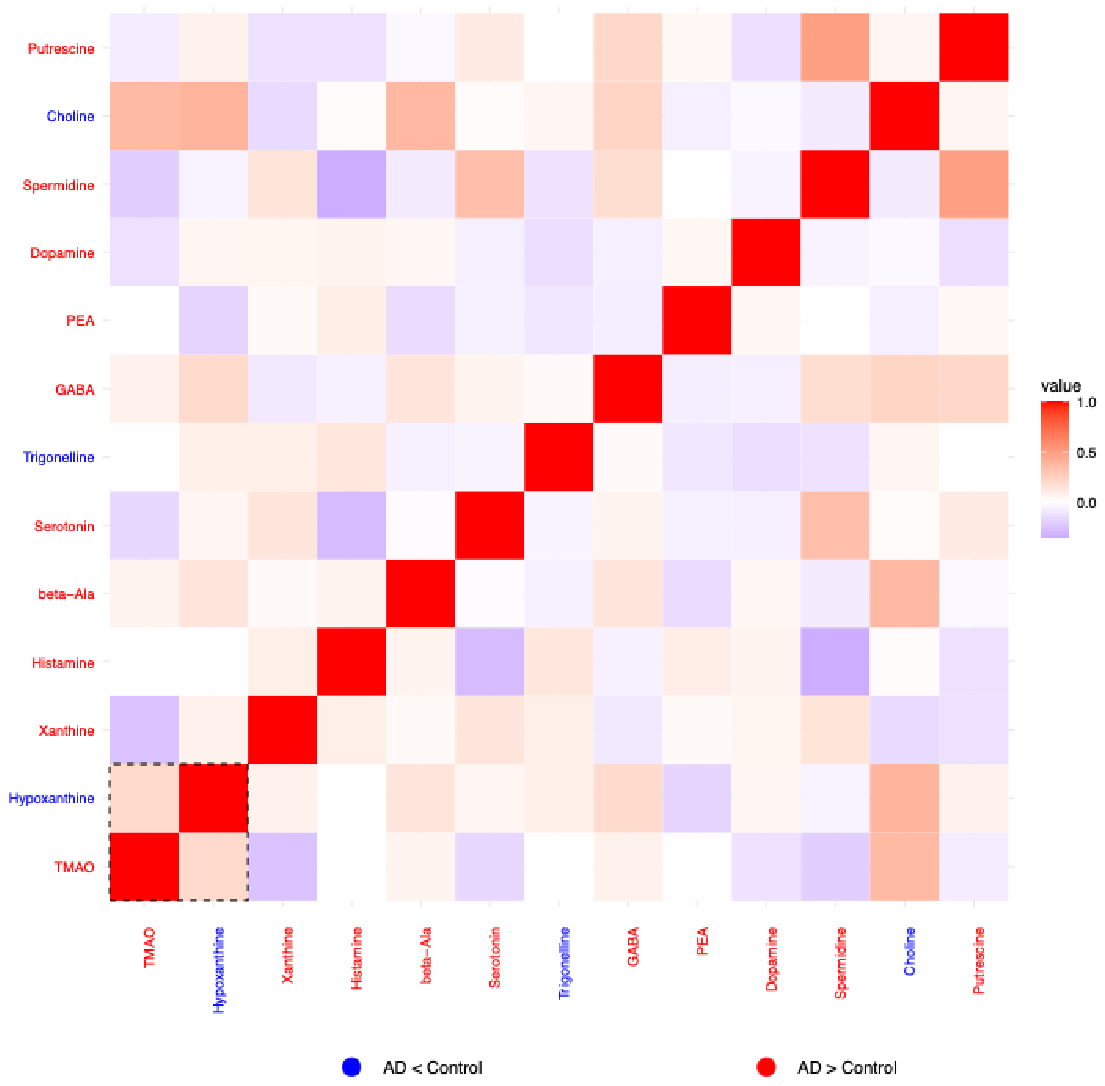

Supplementary Figure S1. Heatmap of correlation of residuals of biogenic amines on comparing data from AD patients and controls. *Metabolite_c* for this family was spermine. Blue labels indicate tendency to decrease in AD and red labels indicate a tendency to increase in AD. Red cells indicate positive correlation (1 means perfect correlation), blue cells indicate negative correlation, and white cells indicate no correlation. $p < 0.05$ for residuals of metabolites within the dashed line frame.

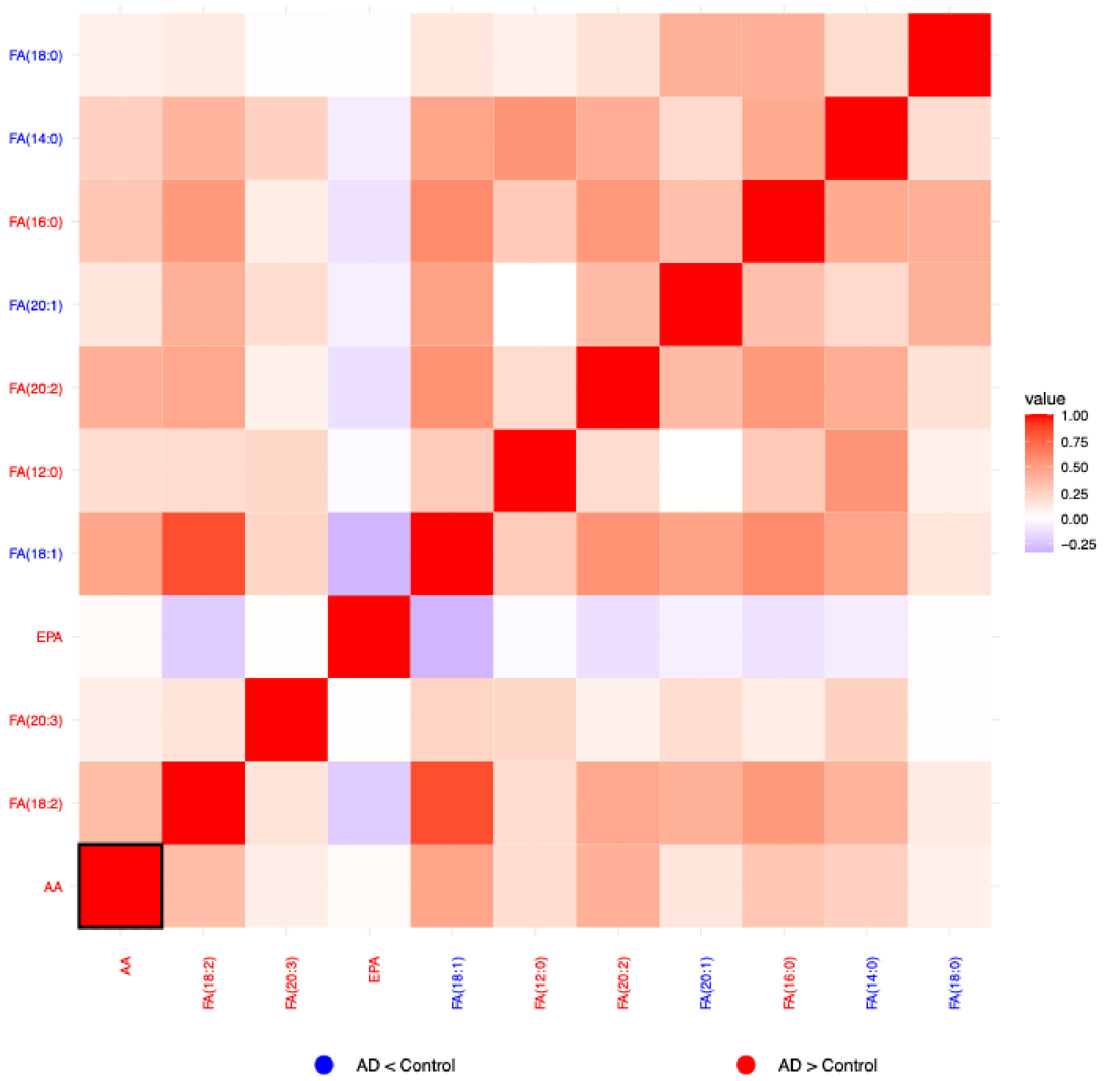

Supplementary Figure S2. Heatmap of correlation of residuals of fatty acids on comparing data from AD patients and controls. *Metabolite_c* for this family was Docosahexaenoic acid (DHA). Blue labels indicate tendency to decrease in AD and red labels indicate a tendency to increase in AD. Red cells indicate positive correlation (1 means perfect correlation), blue cells indicate negative correlation, and white cells indicate no correlation. $p < 0.05$ for residuals of metabolites within the dashed line frame. $P_{adjusted} < 0.05$ for residuals of metabolites within the solid line frame.
